# Song Trait Similarity in Great Tits Varies with Social Structure

**DOI:** 10.1371/journal.pone.0116881

**Published:** 2015-02-18

**Authors:** Lysanne Snijders, Jerine van der Eijk, Erica P. van Rooij, Piet de Goede, Kees van Oers, Marc Naguib

**Affiliations:** 1 Behavioural Ecology Group, Wageningen University, Wageningen, The Netherlands; 2 Department of Animal Ecology, Netherlands Institute of Ecology NIOO-KNAW, Wageningen, The Netherlands; Utrecht University, NETHERLANDS

## Abstract

For many animals, long-range signalling is essential to maintain contact with conspecifics. In territorial species, individuals often have to balance signalling towards unfamiliar potential competitors (to solely broadcast territory ownership) with signalling towards familiar immediate neighbours (to also maintain so-called “dear enemy” relations). Hence, to understand how signals evolve due to these multilevel relationships, it is important to understand how general signal traits vary in relation to the overall social environment. For many territorial songbirds dawn is a key signalling period, with several neighbouring individuals singing simultaneously without immediate conflict. In this study we tested whether sharing a territory boundary, rather than spatial proximity, is related to similarity in dawn song traits between territorial great tits (*Parus major*) in a wild personality-typed population. We collected a large dataset of automatized dawn song recordings from 72 unique male great tits, during the fertile period of their mate, and compared specific song traits between neighbours and non-neighbours. We show here that both song rate and start time of dawn song were repeatable song traits. Moreover, neighbours were significantly more dissimilar in song rate compared to non-neighbours, while there was no effect of proximity on song rate similarity. Additionally, similarity in start time of dawn song was unrelated to sharing a territory boundary, but birds were significantly more similar in start time of dawn song when they were breeding in close proximity of each other. We suggest that the dissimilarity in dawn song rate between neighbours is either the result of neighbouring great tits actively avoiding similar song rates to possibly prevent interference, or a passive consequence of territory settlement preferences relative to the types of neighbours. Neighbourhood structuring is therefore likely to be a relevant selection pressure shaping variation in territorial birdsong.

## Introduction

Maintaining contact with conspecifics is important in many animal populations. Territorial animals are usually constrained to frequently closely approach conspecifics and therefore often maintain contact using long-range signals [1,2], such as song in birds [3]. Birdsong has been a key model in studies on territorial behaviour as it is not only important in territorial defence [3,4], but also to establish and maintain relations with neighbours [5,6]. Birds that possess long-term neighbouring territories can be “dear enemies”, showing reduced aggression towards each other to save time and energy, while still remaining potential rivals in competition for space and mates [7]. Neighbouring songbirds can even form alliances to expel common enemies, like predators [8] and conspecific intruders [9]. Although neighbours are known to be able to adjust their singing to each other during direct singing interactions [4,6], little is known about whether territorial male songbirds affect each other’s song traits in more general terms. By understanding how signal traits vary overall, relative to the social environment, we gain greater insight into the potential selection pressures on territorial signal traits.

Territory advertisement [10], along with inter-sexual advertisement [3,11], is a well-established function of birdsong. For example, in great tits (*Parus major*) [10] and song sparrows (*Melospiza melodia*) [12] broadcasting songs with speakers from inside a territory of which the owner has been removed delays intrusion of the territory by conspecifics. For many songbirds, dawn is a very active signalling period with many individuals singing at the same time, especially during the breeding season [11,13] when intruder pressure is high [14]. To repel potential intruders it is thus important for territory holders to frequently sing during dawn [15,16]. However, singing concurrently with similar song traits compared to immediate neighbours could lead to unintentional continuous song overlapping and song matching. For example, if several neighbours frequently sing concurrently with the same song rate, there is the potential that a part of a neighbour’s song will be unintentionally but continuously overlapped. This is undesirable, because systematic overlapping is often interpreted as an aggressive signal, eliciting strong responses by opponents [3,4,17,18]. Additionally, individual recognition through acoustic signals [5,19], for example by listening females [20], might be compromised when using the same song traits as immediate neighbours. This is important, as immediate neighbours, can pose a high risk for males to lose paternity [7,20]. Either way, in the absence of an immediate and direct conflict, such as between familiar neighbours during regular advertisement singing at dawn [3,13], neighbours might benefit by not singing too similarly [21].

Individual song traits can be predictable when they are to some extent repeatable [22]. However as long as the song traits are not perfectly repeatable, there can still be enough flexibility for slight adjustments, for instance at the start of the breeding season. In great tits, several song traits such as the proportion of time spent singing [23] and song consistency [24] are found to be moderately repeatable. Consequently, territorial great tits could anticipate the repeatable song traits of familiar immediate neighbours and moderately adjust their own song traits in order not to sing too similarly and so optimize signalling towards potential intruders and other eavesdropping conspecifics. In the breeding season, multiple neighbours will often be singing simultaneously for several weeks during dawn, and it would thus be very demanding for individuals to constantly dynamically adjust their singing behaviour to multiple individuals. Moderate repeatability of song traits would allow individuals to optimize signalling among neighbours by adjusting their song traits only a small number of times, for example during the beginning of the breeding season when new neighbours settle.

The aim of this study is to reveal whether or not song traits vary in relation to the song traits of neighbours on a population-wide scale. We therefore recorded the song of 72 unique male great tits during dawn, and examined whether song rate and start of dawn song differed more between immediate neighbours (sharing a territory boundary) than between non-neighbours. Next, we checked whether potential song trait differences between neighbours were not merely a consequence of avoiding overall signal interference. Immediate neighbours are usually singing spatially closer to each other and consequently could be hindering each other’s signal transmission more than non-neighbours. Therefore we also analysed whether differences in song traits were related to the spatial proximity between the breeding nest boxes of the singing males.

## Methods

### Study population

The study was conducted in a long-term study population of great tits at Westerheide, The Netherlands (5°50’E, 52°00’N). Westerheide is a mixed pine-deciduous wood with about 200 nest boxes attached to trees distributed within a 1000 m × 1200 m area (for further details see [25]). Birds in this population are tested for exploration behaviour, an operational measure for personality, using an established and validated ‘novel environment’ test [25,26], approved by the Institutional Animal Care and Use Committee: the Koninklijke Nederlandse Akademie van Wetenschappen—Dier Experimenten Commissie (KNAW-DEC licence NIOO 10.05 to MN and KVO). Exploration behaviour in great tits has been found to relate to social behaviour in wild great tits, both spatially [27] and vocally [28]. Throughout the year, with the exception of the breeding season, newly caught individuals (captured either by mist-netting during the day or during a nest box check at night) are subjected to a novel environment test. Within 1.5 h of catching the great tits are brought to the testing facilities of the Netherlands Institute of Ecology (max 20 min by car), where they are housed individually and undergo the novel environment test the following morning (see [25] for details). The birds were released again within 24 hours after initial capture.

During the breeding season, male great tits sing very actively just before sunrise and maintain high levels of singing activity throughout the pre-laying, laying and incubation period of their mate [29]. Nest boxes were checked twice a week to record laying date (of the first egg), clutch size and hatching date. Following an established procedure, individuals in this study were identified during the chick rearing phase (when nestlings were approximately 7 days old) by catching them inside their nest boxes using spring traps [30]. We took biometry measures of the nestlings when they were 14 days old (with day 0 as hatching date). We measured both body mass and tarsus length on chicks and adults, and un-ringed birds were fitted with uniquely numbered aluminium leg rings.

### Song recordings

Dawn song was recorded once for 72 males during their mate’s fertile period between 1^st^ April and 3^rd^ May 2012 (S1 Table). For 45 of these individuals we recorded dawn song twice during the fertile period; once during early egg laying (1–4 eggs) and once during late egg laying (5–9 eggs), with a mean interval of 4 days (S2 Table). Female songbirds are fertile from approximately a few days before the start of egg laying until the day of the last egg [31]. During the egg laying period, male great tits sing near the breeding nest box of their mate [32]. Therefore, we hung programmable song recorders (Song Meters, Wildlife Acoustics or TASCAM DR-08) 1.5 m above nest boxes occupied by incubating females, the day before recording. Song recordings ran automatically from 1 h before sunrise to half an hour after sunrise.

We determined the start time of dawn song (the time before sunrise when the subject sings their first song) and calculated song rate (songs per minute) during the first 5 min using SASLab Pro (Avisoft, Berlin, Germany). For song recordings of lower quality only the start time was determined. We included males in our analysis only if we had recorded at least one of their five closest surrounding occupied nest boxes, in order to not bias our analyses with individuals who would only be compared to other males at far distances and to males with whom they did not share a territory boundary. Song rate was measured as number of songs per minute (mean = 9.1 songs, sd = 3.0, range = 1.6–16.6) for 67 unique individuals, since for 5 recordings we could not accurately determine the song rate. Start time was quantified as song onset in minutes before sunrise (mean = 31.2, sd = 7.3, range = 4.0–42.2) for 72 unique individuals. With the exception of the repeatability analysis, we always used the first available recording of a nest box for analysis (which was during early egg laying, except for three nest boxes), unless the quality of the first recording did not allow its use (N = 2).

### Environmental variables

Sunrise, moonrise and moonset time data was retrieved from the KNMI (Koninklijk Nederlands Meteorologisch Instituut) and temperature data from the nearest KNMI weather station, Deelen (10 km). Percentage of moonlight, the fraction of the moon’s surface illuminated (arcsine square root transformed), was taken as zero when the moon would not be above the horizon 43 min before sunrise (the earliest singing bird started 42.2 min before sunrise). We calculated distances between breeding nest boxes using GPS coordinates (Garmin GPSmap 62st). Subjects were on average 0.543 km (sd = 0.280 km) apart, with a minimum distance of 0.033 km and a maximal distance of 1.264 km.

### Statistical analysis

For the 45 males we recorded twice during the fertile period of their mate, repeatability of song rate and start time was determined following Lessells and Boag [33]. The range of days between recordings was smaller within individuals (3–6 days) than among individuals (0–32 days), raising the possibility that repeatability in song traits could be merely a reflection of more similar environmental conditions caused by the shorter time between recordings. To test this, we calculated the within—and among-days variation in song rate and start time of dawn song for both early and late egg laying, only including days on which more than one bird was recorded. Within-day variation in song rate was not smaller compared to among-day variation in song rate (Early egg laying, *F_11,21_* = 0.99, *p* = 0.48; Late egg laying, *F_11,22_* = 0.89, *p* = 0.56) and also within-day variation in start time of dawn song was not smaller compared to among-day variation in start time (Early egg laying, *F_11,21_* = 0.83, *p* = 0.62; Late egg laying, *F_11,22_* = 1.68, *p* = 0.14). Additionally, we conducted General Linear Mixed Models (GLMM) using SPSS 21.0 (IBM Corp., Armonk, NY, USA) to test for a possible linear effect of April date on song rate and start time of dawn song, including individual as a random factor to account for multiple measurements of the same individual. Residuals of both models followed a normal distribution, based on the Shapiro-Wilk test. We tested for a possible association between the condition of the bird and song rate or start time using a Pearson correlation test. Individual condition was calculated as the residual of the function of weight (body mass during chick rearing) against tarsus. We analysed whether there was a difference in song traits between one-year-old males and birds older than one year (2 years or older) using an Approximative Wilcoxon Mann-Whitney Rank Sum Test (10 000 re-samplings) from the *Coin* R-package. Additionally, median song trait differences for one-year-old males were bootstrapped with the *boot* R-package to generate confidence intervals and so account for the difference in sample size, compared to the males older than one year, which could lead to spurious significant results. For all subsequent analyses we used R 2.15.2 (R Core Team, Vienna, Austria).

We calculated differences in song rate and start time between males for all comparisons (2211 ((67*66)/2) for song rate and 2556 ((72*71)/2) for start time; S3 Table). We conducted a tessellation using the *AdeHabitat* R-package. The Voronoi/Dirichlet tessellation procedure systematically divides the habitat into convex polygons (territories), each centred around the breeding nest box of a territorial male great tit. Each great tit territory has the property that every point within the polygon (territory) is nearer to its own nest box than it is to any of the other nest boxes. Birds were categorized as neighbours if they shared part of their territory boundary and their nest boxes were within 242 m of each other (twice the maximum estimated un-manipulated mean territory size [34]). To account for the dependence of data when testing differences in similarity between neighbours and non-neighbours, we employed the Approximative Wilcoxon Mann-Whitney Rank Sum Test (10 000 re-samplings) from the *Coin* R-package. Additionally, median song trait differences for non-neighbours were bootstrapped with the *boot* R-package to generate confidence intervals and so account for the difference in sample size compared to the neighbour dataset.

Because we expected that a relationship between space and song traits would unlikely be linear, but fade with increasing distance around several centres in the population (like ripples caused by drops falling into water), we specifically tested for non-linear effects using several distance classes [35]. The *Ecodist* R-package tests for (dis)similarity of a certain feature within several distance classes (lags), by calculating a Mantel correlogram, a multivariate autocorrelation function. The *Mantel r* represents the (dis)similarity (of individual song trait in this study) at a certain lag distance. We used six distance lags, which are classes dividing the distance range between all possible pair combinations in six groups of equal distance intervals, ranging from close together (lag 1, midpoint 0.103 km) to exceedingly far apart (lag 6, midpoint 1.128 km). However, we only considered the first four lags when drawing conclusions, since an increasing number of birds was no longer represented in the last two lags (from 0.666 km onwards). We chose the number of six lags *a priori*, since a lower number would have resulted in too many distant pair combinations being included in the first (closest) distance lag (the closest pair combination was 0.033 km apart), and a higher number would have led to an increasingly smaller sample size for the closest distance lag. Increasing the number of lags (to 8 or 10) did not significantly change the outcome of the analysis.

When permutation or randomization procedures were employed, significance levels are shown using “<” or “>”, since in permutation models values for significance are based on approximation.

## Results

### Are great tit dawn song traits repeatable?

**Figure 1:**
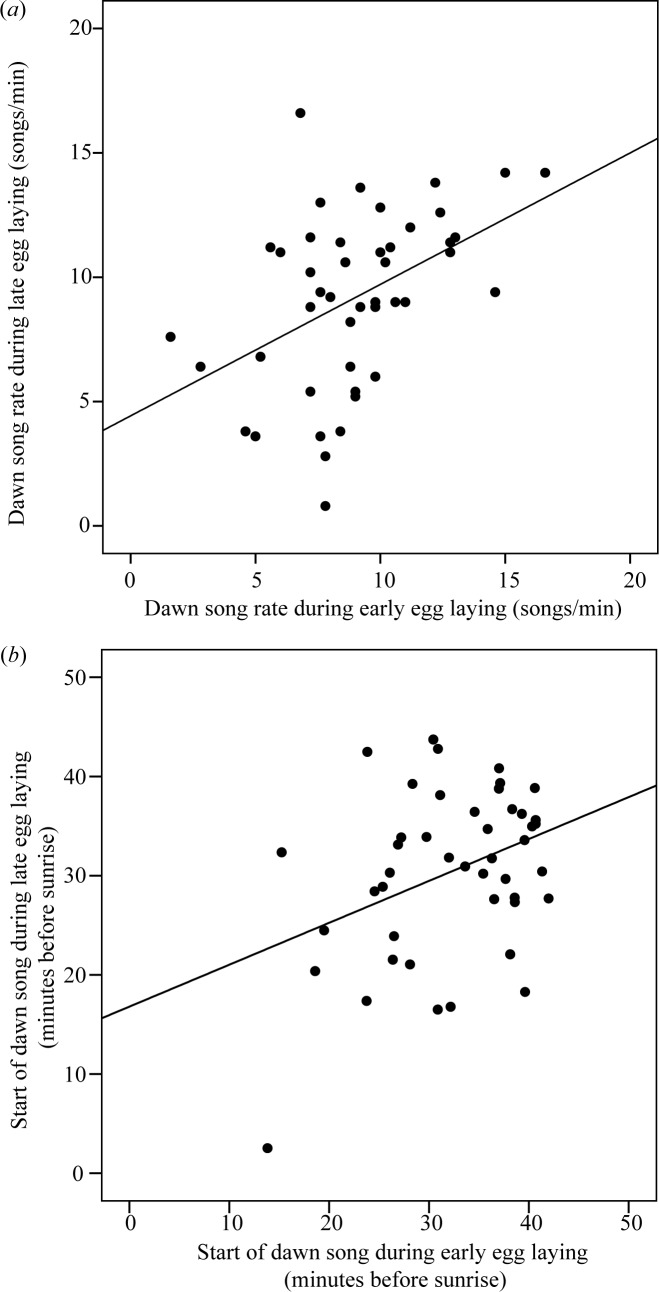
Male great tits are significantly repeatable in (*a*) song rate as well as (*b*) start time of dawn song. The song of 45 individual great tits was recorded twice in the fertile period of their mate, first during early egg laying (1–4 eggs) and second during late egg laying (5–9 eggs). Differences in these dawn song traits among individuals were significantly larger than within individuals.

The song rate of individual birds was significantly moderately repeatable (*r* = 0.45, SE = 0.12, *F_44,45_* = 2.67, *p* = 0.001; Fig. 1A), as was the start time of dawn song (*r* = 0.36, SE = 0.13, *F_44,45_* = 2.10, *p* = 0.007; Fig. 1B). However, it should be noted that repeatability of start time decreased (*r* = 0.21, SE = 0.14, *F_43,44_* = 1.54, *p* = 0.078), when leaving out the male with the latest start time (2.5 minutes before sunrise during late egg laying). There was no linear relationship between date and song rate (GLMM, *F_1,44_* = 0.21, *p* = 0.65) or start time of dawn song (GLMM, *F_1,44_* = 1.90, *p* = 0.18). Additionally, there was no correlation between body condition and song rate (Pearson *r* = 0.08, *n* = 41, *p* = 0.63) or start time (Pearson *r* = −0.13, *n* = 42, *p* = 0.42). Finally, there was also no difference in song rate between one-year-old males and males older than one year (*Z* = −0.89, *n1* = 40 and *n2* = 13, p>0.3), but there was a difference in start time of dawn song, with younger males starting to sing earlier than older males (*Z* = 2.19, *n1* = 43 and *n2* = 15, p<0.05). But this significant effect of age was not reflected by the bootstrapped confidence intervals as the median start time of the older males (28.4 minutes before sunrise) did still fall within the 95 percentage bootstrapped confidence interval for the younger males (*CI* = 26.9–37.2 min before sunrise; drawing 15 samples 10 000 times without replacement).

### Does song trait dissimilarity correlate with sharing a territory boundary?

**Figure 2:**
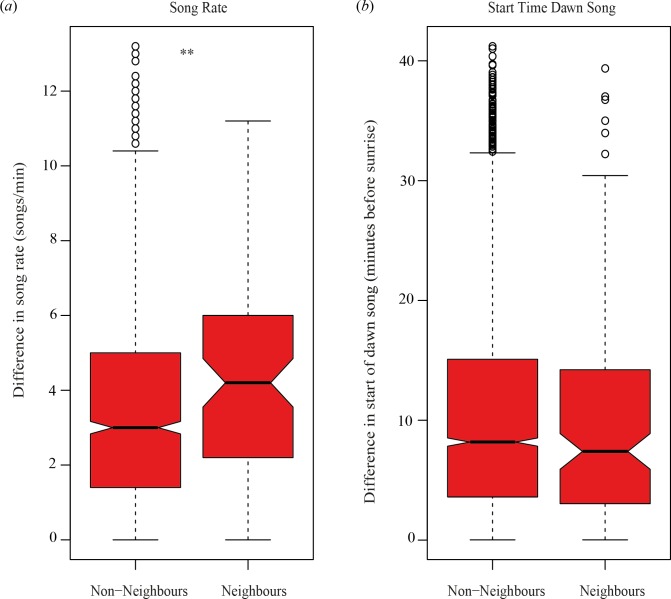
Notched box-plots reveal that (*a*) neighbours (birds sharing a territory boundary) differ more from each other in median song rate, (*b*) but not in median start time than non-neighbours do. Non-overlapping notches visualise a significant difference in medians.

Neighbouring birds (114 out of 2211 pair combinations) were significantly more dissimilar in song rate than non-neighbouring birds were (*Z* = −2.66, *p*<0.01; Fig. 2A). The median difference in song rate for neighbours was 3.3 songs/min, falling outside the 95 percentage bootstrapped confidence interval for non-neighbours (*CI* = 2.2–3.2 songs/min; drawing 114 samples 10 000 times without replacement). Neighbours (123 out of 2556 pair combinations), however, were not more similar or dissimilar in start of dawn song compared to non-neighbours (*Z* = 1.24, *p*>0.2; Fig. 2B). The median difference in start time for neighbours was 6.4 min before sunrise, falling inside the 95 percentage bootstrapped confidence interval for non-neighbours (*CI* = 6.0–9.2 min before sunrise; drawing 123 samples 10 000 times without replacement).

### Does song trait dissimilarity correlate with spatial proximity?

We found no evidence for a non-linear relationship between breeding distance and song rate, since birds that bred closest together did not significantly differ in song rate (Lag 1, midpoint: 0.103 km, 268 pair combinations, *Mantel r* = −0.02, *p*>0.3) and there was only a trend for birds breeding very distant from each other to be more dissimilar in song rate (Lag 4, midpoint: 0.718, 419 pair combinations, *Mantel r* = −0.04, *p*<0.1). However, birds breeding closest together (Lag 1) did have significantly more similar start times of the dawn song, while birds breeding one additional distance lag apart (Lag 2) did not have more similar start times (Table 1). Additionally, birds further away from each other (Lag 3) differed significantly in start time, while there was no significant relation between birds even further apart (Lag 4; Table 1).

**Table 1:** Non-linear spatial analysis of start time of dawn song similarity (minutes before sunrise) and temperature similarity (decimal degrees) using distance classes (lags).

		**Lag 1**	**Lag 2**	**Lag 3**	**Lag 4**
Start time of dawn song similarity	*Mantel r*	0.039	−0.04	−0.07	0.01
	*p*	**<0.05**	>0.1	**<0.05**	>0.5
Temperature similarity	*Mantel r*	0.09	−0.01	−0.05	−0.10
	*p*	**<0.001**	>0.7	<0.08	**<0.001**
Moonlight similarity	*Mantel r*	0.02	−0.001	−0.01	−0.05
	*p*	**<**0.23	>0.9	>0.6	**<0.05**
	*Midpoint (km)*	0.103	0.308	0.513	0.718
	*N*	309	600	697	460

There was also no linear relationship between the breeding distance and the difference in song rate between males (*Mantel r* = −0.05, *p*>0.8). When controlling for breeding distance, neighbours still tended to differ more in song rate than non-neighbours (Partial Mantel with Non-Neighbours and Neighbours, *Mantel r* = 0.04, *p*<0.06). Likewise, there was no linear relationship between breeding distance and difference in start time (*Mantel r* = −0.05, *p*>0.8) and when controlling for breeding distance, neighbours were not significantly similar or dissimilar in start time of dawn song compared to non-neighbours (Partial Mantel with non-neighbours and neighbours, *Mantel r* = −0.04, *p*>0.9).

### Could dissimilarity in song traits be explained by dissimilarity in personality or environmental conditions?

Neighbours (75 out of 1225 pair combinations) were not more similar or dissimilar in exploration behaviour compared to non-neighbours, for either song rate analysis (*Z* = 0.33, *p*>0.7) or start time analysis (80 out of 1431 pair combinations, *Z* = 0.53, *p*>0.5). However, although pairs of neighbours were not more similar in laying date (*Z* = 1.34, *p*>0.1), pairs of neighbours in the analysis of song rate did have fewer days between recordings than non-neighbours (*Z* = 2.05, *p*<0.05). Likely because of the shorter time between recordings, pairs of neighbours were also recorded during more similar temperatures compared to non-neighbours (*Z* = 4.69, *p*<0.001). Neighbour recordings were not more similar, or dissimilar, in moonlight conditions compared to non-neighbours (*Z* = 0.19, *p*>0.8). Still, similarities in environmental conditions during recordings of neighbours compared to non-neighbours cannot explain, and are in contrast to, the significant dissimilarity of song rate between neighbours.

For the analysis of start time, the closest breeding birds were more similar in laying date (Lag 1, midpoint: 0.103 km, 309 pair combinations, *Mantel r* = 0.05, *p*<0.01) and were recorded with fewer days in between (Lag 1, midpoint: 0.103 km, 309 pair combinations, *Mantel r* = 0.06, *p*<0.005), while there was a trend for very distant birds to be more dissimilar in laying date (Lag 4, midpoint: 0.718 km, 460 pair combinations, *Mantel r* = −0.04, *p*<0.1) and to be recorded with more days in between (Lag 4, midpoint: 0.718 km, 460 pair combinations, *Mantel r* = −0.05, *p*<0.05). Hence, likely because of fewer days between recordings, the birds breeding closest to each other (Lag 1) were also recorded with more similar temperatures, while very distant birds (Lag 4) were recorded with significantly dissimilar temperatures and dissimilar moonlight conditions (Table 1). Yet, we did not find a linear relationship between similarity in start time and similarity in laying date (*Mantel r* = −0.01, *p*>0.5), temperature (*Mantel r* = −0.02, *p*>0.5) or moonlight (*Mantel r* = −0.01, *p*>0.5).

## Discussion

Our analysis of dawn song characteristics revealed that great tit song rate and start time during dawn song were significantly moderately repeatable, allowing neighbours to anticipate and respond by adjusting their own song traits accordingly [22]. Indeed, we found that sharing a boundary significantly related to the similarity in song rate of birds, in which neighbours differed more in song rate when compared to non-neighbours. We did not find an effect of neighbours on start time of dawn song, however we did find that males breeding close together started dawn song at more similar times than males breeding further apart, who started at more dissimilar times.

### What could drive dissimilarity in song rate between neighbours?

The dissimilarity in song rate between neighbours could result from: (1) an active process of neighbouring males deliberately adjusting their song traits or (2) a passive process in which song rate differences result from different types of males settling nearby.

Neighbour-related differences in singing could result from familiar neighbours anticipating and actively averting the potential for unintentional continuous overlapping of each other [36]. By singing at different rates, males could be able to reduce signal interference and advertise territorial ownership without risking of being perceived as aggressive by their neighbours and so escalate an interaction [4]. For instance in black-capped chickadees (*Poecile atricapillus*) individuals breeding in the same neighbourhood frequency match each other (sang song types with similar frequencies) to a lesser degree when they are familiar with each other (in the same winter flock the previous winter) [6]. Additionally, in this unique study using an acoustic location system to record dawn singing interactions, Foote *et al*. [6] also did not find an effect of neighbour proximity on the degree of matching, comparable to the lack of an effect of proximity on song rate that we found. A promising avenue for future work is further large-scale studies to examine if and how often similarity in song rate leads to consistent overlapping by concurrently singing neighbours and whether this would elicit aggressive interactions between them. Moreover, we recently showed that territorial male great tits encounter each other non-randomly [27], raising the question to what extent these social, possibly aggressive, encounters relate to the non-random variation in song trait differences we revealed here.

Besides avoiding neighbour aggression, singing distinctly from neighbours might also function in affecting behaviour of mates, who in song birds have been shown to use song information in extra-pair mating decisions [37]. Especially since the great tit is a socially, but not sexually, monogamous species [30], it might be very relevant for males to distinguish themselves from neighbours using their song traits. Moreover, the difference in song rate could also be a consequence of neighbours distinguishing themselves by singing with different song types (that differ in duration), as individual male great tits can sing up to approximately seven different song types [38]. However, great tit males in close proximity actually tend to be more similar in their total song repertoire [39] and songs added to the song repertoire of individuals were found to mostly resemble, not differ from, those of newly arrived neighbours [40].

Here we show that song rate varies in relation to specific social relationships between territory owners, i.e. whether or not territory owners share a territory boundary, but next to active social processes our findings could also result from a more passive process. Song traits often link to individual quality, like age or condition [41,42], hence neighbour-related differences in song might also be the result of territorial birds preferring to settle next to individuals that differ from themselves to increase contrast and recognisability [13,28], to reduce (mate) competition [43] or to form complementary alliances [9]. We did not find neighbours to be more dissimilar in personality (exploration behaviour) than non-neighbours and there was no linear correlation between song rate and body condition, or any difference in song rate between young and older males. Although differences in personality, age and body condition could not explain the difference we found in the song rate between neighbours compared to non-neighbours, we cannot rule out the possibility that other (unmeasured) traits related to individual quality, like dominance rank [44], might explain the neighbour dissimilarity in song rate.

However, even when song traits are found to be honest indicators of individual quality, it does not rule out that there is still flexibility for birds to also moderately adjust these song traits in response to social stimuli. For example, in black-capped chickadees, both song rate and start of dawn song were found to be honest indicators relating to dominance rank [44], yet territorial black-capped chickadees were still able to moderately advance their start time when provoked by a simulated intruder [45]. Both the active and passive scenarios discussed above imply a significant role for neighbours in signalling, and an experiment in which the song rate of neighbours is manipulated could give valuable insight in the actual underlying mechanism.

### What could drive similarity in start time of dawn song between males breeding close together?

In contrast to our findings on song rate, our results suggest that start time of dawn song is less influenced by specific social relations (sharing a boundary or not), and more by proximity. However, birds breeding close together were also recorded at more similar temperatures, which could have partly driven the spatial pattern found. In addition to sunrise [3], start time often associates with variable environmental factors like lunar phase [46], night temperature [47] and in some cases also noise levels [48]. This might also explain why the strength of the correlations with space, though significant, were rather small (*Mantel R between −0.1 and 0.07)*. Yet, seasonal environmental factors are unlikely to explain the specific spatial pattern in this study, as within-day variation in start time of dawn song was not larger than among-day variation and there was no linear relation between start time similarity and temperature or moonlight similarity.

Alternatively, the spatial pattern we found could be caused by a social contagion effect, the spread of behaviour patterns in a group through imitation and conformity, or facilitation effect [49,50]. The start of dawn song by one bird could trigger nearby conspecifics to also start singing, while conspecifics further apart from this starting bird will respond to other, relatively closer, starting birds, creating a specific non-linear wave-like pattern of similarity and dissimilarity between close and more distant birds. This is in line with a previous study on nightingales (*Luscinia megarhynchos*) showing indeed that song originating from outside the territory triggers strong singing responses [51] and that less degraded (near) song leads to stronger responses than more degraded (distant) song [52]. Also a similar pattern was found in territorial chaffinches (*Fringilla coelebs*), in which the degree of similarity in singing activity decreased with distance in space [53]. Additionally, neighbouring black-capped chickadees slightly, but significantly, advanced the start of their dawn chorus after on the same morning a simulated unknown intruder started singing firstly from within a neighbour’s territory [45].

Apart from social contagion, similarity in habitat structure and food availability [15,54] for birds breeding closer together could also explain the spatial effect on start time in great tits; for example if breeding near forest edges allows more natural and artificial light to penetrate during dawn [55] and if spatial variation in local food availability during dawn affects the optimal time to start foraging [15]. The combination of both habitat structure and social contagion to be driving the spatial pattern is also a possibility. The repeatability of start time implies that the same individuals generally start singing first, creating a relatively consistent order of starting males as was found in black-capped chickadees [45], which could consistently set off the rest of the neighbourhood.

Yet, as with the dissimilarity in song rate between neighbouring birds, similarity in start of dawn song could potentially be caused by non-random territory settlement when certain song traits relate to certain other individual characteristics [41] [42]. Although we did not find a correlation between start time and body condition, we did find indications that younger males start their dawn song earlier than older males, which could explain the spatial pattern if males of similar age settle closer together. However it is more likely that first year males settle in between older (resident) males, because site fidelity combined with age- and site-dependent dominance will presumably favour males from the previous year to win local disputes against other males in the process of establishing a breeding territory [56], creating a mixed spatial pattern of old and young birds.

Future population-level studies of territorial bird song analysing the start time of dawn song on similar days are necessary to support the social contagion hypothesis for territorial great tits. Additionally, future studies on bird song traits incorporating both habitat and social structure [57] would be insightful to disentangle both of these possible selection pressures.

### Concluding remarks

Our findings shed a new light on our understanding of communication networks, as they suggest that neighbours, and not solely spatial proximity, have a significant role in the use of signal space in territorial birdsong. Individual song is often part of a larger communication network and future studies on birdsong should thus take this social and spatial structure into account.

## Supporting Information

S1 Table Individual Song Traits. Individual characteristics and environmental conditions during the time of song recording. *Nest box*: Number of the nest box were the song recorder was placed, *Exploration score*: Exploration score for the breeding male caught in the nestbox; *Age*: One-year old male (= 1) or older (= 2), *Condition*: The unstandardized residual of the linear regression of mass over tarsus (for analysis only condition for weights measured after 12:00 were used), *First egg date*: Date of the first egg laid by the breeding female in April days (April 1st = 1), *Recording date*: Date of song recording counting in April days (April 1st = 1), *Start time*: Start time of dawn song in minutes before sunrise, *Song rate*: Song rate of dawn song in number of songs per minute (first 5 minutes), *Temperature*: Temperature during the time of song recording in decimal degrees Celcius, *Moonlight*: Proportion of moonlight during the early morning of the song recording (arcsine square root transformed and taken as “0” when the moon was below the horizon during dawn)
(XLSX)

S2 Table Repeatability Song Traits. Individual song characteristics of birds included in the song repeatability analysis. *Nest box*: Number of the nest box were recording took place, *Recording day 1*: Date of recording counting in April days (April 1st = 1), during early egg laying, *Start time*: Start time of dawn song in minutes before sunrise, during early egg laying, *Song rate*: Song rate of dawn song in number of songs per minute (first 5 minutes), during early egg laying, *Recording day 2*: Date of recording counting in April days (April 1st = 1), during late egg laying, *Start time 2*: Start time of dawn song in minutes before sunrise, during late egg laying, *Song rate 2*: Start time of dawn song in minutes before sunrise, during late egg laying
(XLSX)

S3 Table Dissimilarity Song Traits. Information for all possible male-to-male comparisons on dissimilarity in individual characteristics, dissimilarity in song recording conditions, nest box distance and the presence or absence of a shared territory boundary. *Nest box pair*: The number of the nest boxes of the breeding males who were compared, *Start time difference*: Difference between the two breeding males in the start time of dawn song in minutes, *Song rate difference*: Difference between the two breeding males in the song rate in number of songs per minute, *Neighbour*: If the breeding males shared a part of their territory boundaries with eachother (= 1) or not (= 0), *Distance*: The distance in km between the nest boxes of the breeding males, *Exploration difference*: Difference between the two breeding males in exploration score, *Recording day difference*: Number of days between the song recordings of the two breeding males, *Egg laying difference*: Number of days between the dates the first egg was laid by the mates of the two breeding males, *Temperature difference*: The difference between the two breeding males in the temperature in decimal degrees Celcius during the time of recording, *Moonlight difference*: Difference between the two breeding males in the proportion of moonlight during the early morning of the recording (arcsine square root transformed and taken as “0” when the moon was below the horizon during dawn)
(XLSX)
